# Time of self-harm presentations to hospital emergency departments: a scoping review

**DOI:** 10.1007/s00127-022-02353-4

**Published:** 2022-09-02

**Authors:** David Mc Evoy, Mary Clarke, Mary Joyce

**Affiliations:** 1 Population Health and Health Services, Royal College of Surgeons Ireland (RCSI), Beaux Lane House, Mercer Street Lower, Dublin 2, Ireland; 2 Department of Psychiatry, Royal College of Surgeons in Ireland, Education and Research Centre, Beaumont Hospital, Dublin 9, Ireland; 3 National Suicide Research Foundation (NSRF), Western Gateway Building, University College Cork, Cork, Ireland

**Keywords:** Self-harm, Self-injury, Emergency department, Presentation time

## Abstract

**Background:**

The time at which a self-harm presentation occurs has been shown to be a significant factor as to whether a patient receives a psychiatric assessment or not, which may benefit the patient’s future care. This scoping review sought to identify studies that report on the peak time of day for self-harm presentations to hospital Emergency Departments (EDs). This could help hospital managers to properly allocate the appropriate services for self-harm patients when they are needed the most.

**Methods:**

A scoping review of the literature from the year 2000 until 30th June 2021 was carried out using the PubMed, Web of Science, Embase and the Cochrane library databases.

**Results:**

There were 22 studies that were included for data extraction. The findings from 20 of these studies indicate that self-harm presentations tend to occur outside of working hours (09:00–17:00, Monday to Friday). The majority of studies found that the peak time for self-harm presentations was in the hours before and after midnight.

**Conclusions:**

While this scoping review identified a satisfactory number of studies for data extraction, examination of time of day of presentation was a secondary outcome across most studies. Given that the majority of studies focused on adult samples, further research is necessary to investigate peak times for other age cohorts. More research on this topic is also needed in low- and middle-income countries. Consideration should be given to ensure that the necessary resources to treat hospital presenting self-harm are allocated outside of typical working hours.

## Introduction

Suicide and self-harm are major global health problems. According to the World Health Organization (WHO), more than 700,000 people die by suicide worldwide each year [1]. Suicide affects all age groups but has a particularly high rate among adolescents and young adults, and is the fourth leading cause of the death in 15–29 year olds [2]. Moreover, for every suicide there are many more suicide attempts, and a previous suicide attempt has been identified as the single most important risk factor for suicide in the general population [1]. In the UK, it has been estimated that approximately half of all individuals who die by suicide have a history of self-harm [3].

For the purposes of this study, we used the Platt et al. definition for self-harm; namely, “an act with non-fatal outcome in which an individual deliberately initiates a non-habitual behaviour, that without intervention from others will cause self-harm, or deliberately ingests a substance in excess of the prescribed or generally recognised therapeutic dosage, and which is aimed at realising changes that the person desires via the actual or expected physical consequences” [4]. This definition for self-harm was developed by the WHO/EURO working Group, which replaced the term “parasuicide” with “deliberate self-harm” [5].

Patients who present to hospital following an act of self-harm have been identified as one of the groups at highest risk of suicide [6]. It is therefore critical to study the profile of this group of individuals to identify potential at-risk groups. Surveillance and monitoring systems which monitor the occurrence of hospital-presenting self-harm help to identify at-risk groups and have been established in various areas: for example in Northern Ireland and Manchester [7, 8]. In the Republic of Ireland, the National Self-Harm Registry Ireland (NSHRI) is an example of a national surveillance system of hospital-presenting self-harm, and was the first in the world to achieve national coverage of all Emergency Departments (EDs) in one jurisdiction [5]. One of the benefits of surveillance systems such as the NSHRI is that at-risk groups can be identified and prioritised for treatment. Arensman et al. outline that international guidelines advocate the need for standardised assessment and management procedures for self-harm, yet highlight that many studies have shown that admission rates and assessment procedures vary in different regions, with rates of psychosocial assessment ranging from 36 to 82% [9]. This is evident in a study by Kapur et al. who found that 90% of patients presenting with self-harm had evidence of a psychiatric disorder at the time of presentation, yet they note that only 60% of patients received a psychiatric assessment [10].

Detailed reporting on information about hospital-presenting self-harm can also be utilised by hospital managers and policy makers to assist in the planning and provision of services. An advantage of having current (and where possible real-time) data on self-harm presentations at EDs is that hospital managers can monitor the occurrence and trends of high-risk individuals and allocate services where and when they are needed most. Arensman et al. found that the time of presentation was a significant factor that contributed to patients’ next care; that is, whether they received a psychiatric assessment or not [9]. Indeed, if the most frequent times of self-harm attendances can be determined, then the findings could potentially be used by hospital management teams to allocate adequate services at critical times. Following a literature review to further examine the significance of time of presentation in relation to care of self-harm patients, it is apparent that a gap exists with respect to studies in this area.

With this in mind, we decided to conduct a scoping review to determine what evidence is available internationally about the peak times at which self-harm presentations to EDs occur. Given the potentially limited number of studies which exclusively report on ED presentation times following self-harm, we chose to conduct a scoping review to assess the amount of available evidence on this topic.

## Methods

We followed the PRISMA Extension for Scoping Reviews (PRISMA-ScR) checklist for conducting this study [11]. A review protocol was established for this work but it was not pre-registered as this is a potential precursor to a more refined systematic review and PROSPERO does not register scoping review protocols at present [12].

### Search strategy

**Fig. 1:**
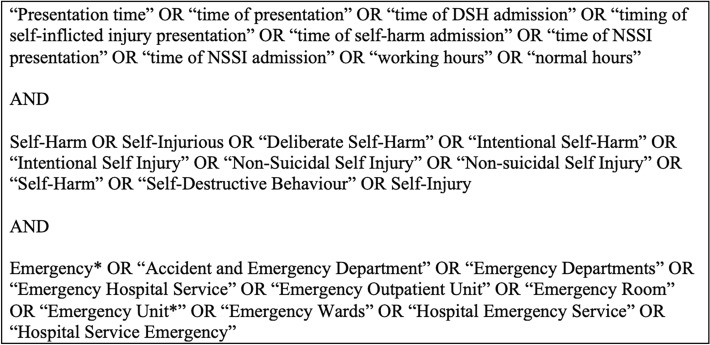
Search terms used in the search strategy

The databases used for the purpose of this review were PubMed, Web of Science, Embase, and the Cochrane library. The search included published articles from the year 2000 up until 30^th^ June 2021. This was to ensure that this study gave a contemporary and up-to-date picture of self-harm presentations in recent times—for which we chose approximately the last twenty years. The search strategy, designed by DMcE and MJ, involved Boolean operators and the wildcard function for terms relating to “presentation time”, “self-harm” and “emergency department”. We examined the literature to establish the terms and phrases relating to these three concepts. Moreover, a librarian from the Royal College of Surgeons Ireland (RCSI) was consulted with respect to the search strategy and search terms. The full format of this search strategy can be seen in Fig. 1. Following this, the results from the mentioned databases were compiled together by DMcE using the free online software Rayyan for screening titles and abstracts for various types of reviews [13].

### Primary screening (eligibility criteria)

A set of inclusion/exclusion criteria was predetermined by DMcE and MJ. Inclusion criteria included peer reviewed studies published in English since 2000 that used point-in-time hospital or registry data of self-harm presentations to EDs. Exclusion criteria included letters, editorials, case studies and case series. Further exclusion criteria included articles relating to prevention of self-harm; articles relating to other diseases not relevant to this study; and, articles concerning other mental health presentations at EDs.

During the primary screening process, DMcE removed any duplicates and screened the titles and abstracts of each study according to the inclusion/exclusion criteria. While the outcome of interest for this study was the peak time of day at which self-harm presentations occurred in EDs, it became evident that this was not usually the primary outcome in the studies reviewed here. Hence, any article that included point-in-time data relating to self-harm presentations to EDs were included for the secondary screening process so that any information pertaining to time of presentation was not inadvertently missed. Only studies relating to self-harm presentations to EDs were included so studies involving self-harm presentations at general practice facilities were also excluded, since general practice usually operates during typical office working hours of 09:00–17:00, Monday to Friday.

### Secondary screening (study selection)

The included studies from the primary (title and abstract) screening then went through a full-paper secondary screening phase (by DMcE in consultation with the other authors). Studies that used data from EDs (or self-harm registry data based on ED data) to provide information on the most common timeframe for self-harm presentations were included for data extraction and analysis.

Six other articles were included for screening [3, 14–18]. Four of these articles [3, 14–16] were known to the first author and one article [18] was known to MJ. The primary outcome for this study, namely time of self-harm presentations at EDs, was a minor outcome mentioned in these five studies and thus the search terms in Fig. 1 did not capture these five articles. The expertise of Dr. Paul Corcoran, from the National Suicide Research Foundation in Ireland, was also sought in relation to any obvious omissions after the search terms yielded the results. Hence, the sixth study, by Corcoran et al., included for screening, was recommended by the first author of that study [17]. These six studies were checked against criteria for the primary and secondary screening process before being included for data extraction.

### Data extraction and quality assessment

The following data was extracted for each study: year of publication; country in which the study took place; the study design; the type of analysis used; the source of the data; the type of data used in the study; self-harm inclusion criteria (the definition of self-harm used in the study); data relating to methods of self-harm; the studied population; notes regarding the times of self-harm presentations at EDs; notes regarding any other secondary outcomes (such as the most common days or months for self-harm presentations at EDs); and, any other notes of interest regarding the study. Each study was also assessed for quality mainly owing to the type and quality of the data obtained in that study.

## Results

**Fig. 2:**
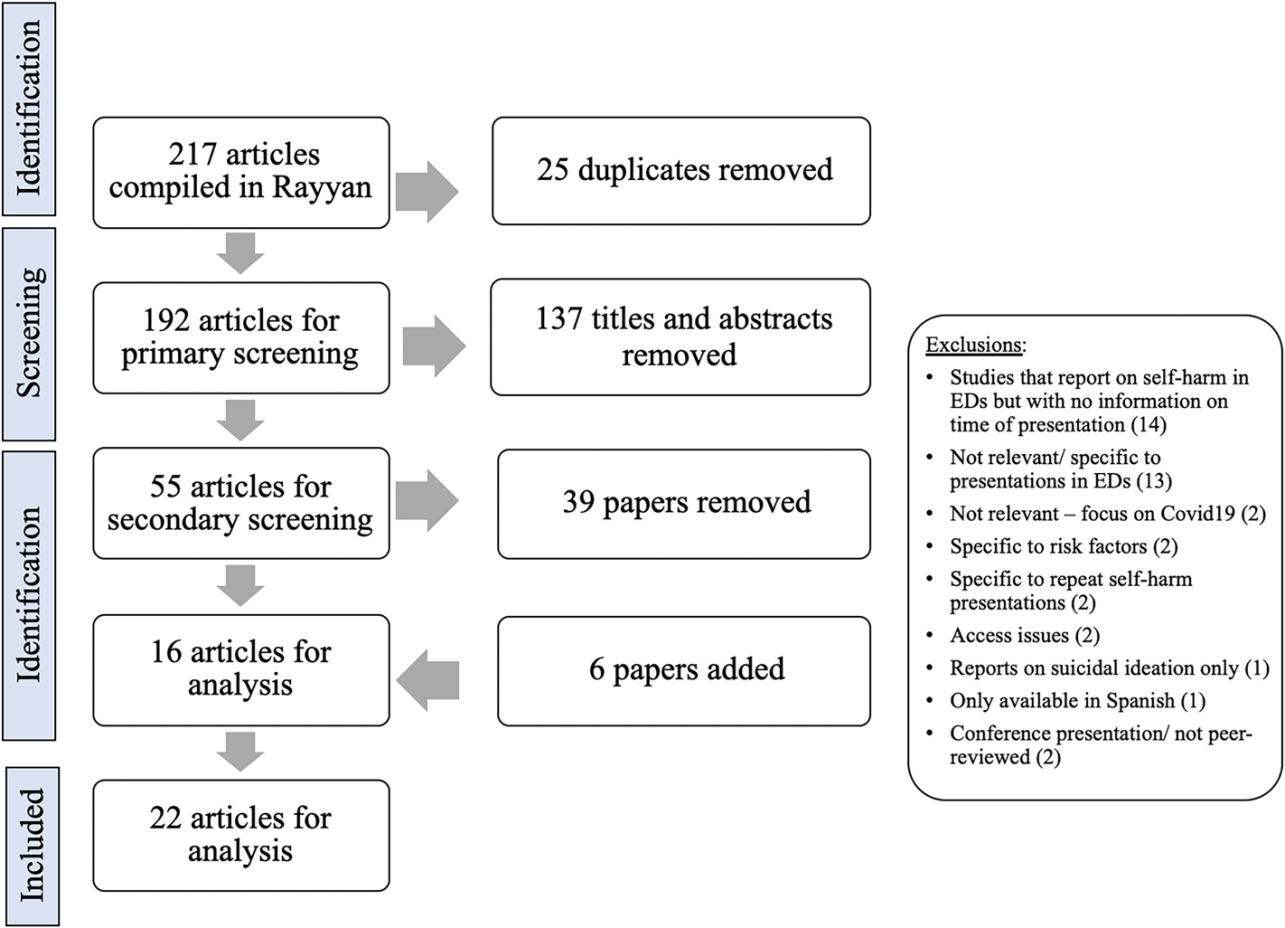
The results from the screening process

There were 217 articles compiled in Rayyan from PubMed (175), Web of Science (13), Embase (25), and the Cochrane library (4). Following this, there were 25 duplicates removed leaving 192 articles for the primary (title and abstract) screening process. There were no relevant reviews (of any type) included during primary screening. During the primary screening process, 137 articles were deemed irrelevant leaving 55 articles for the secondary screening process. Since time of self-harm presentation was not typically a primary outcome in these studies, any study that used point-in-time hospital data of self-harm presentations, cohort data, or that completed descriptive analysis of ED data relating to self-harm were included for secondary screening. This resulted in all 55 studies from the primary screening process proceeding to full-text review by the first author to determine if time of self-harm presentations to EDs was included as an outcome in the study. During the secondary screening process, 39 studies were excluded and 16 studies were included for data extraction and analysis. Six additional studies [3, 14–18]—five of which were known to the authors and one additional study that was recommended by an expert in the area—were then included after being assessed with the same criteria used during the primary and secondary screening processes. Hence there were 22 articles in total for data extraction and analysis. A summary of the process can be seen in Fig. 2.

The characteristics of each study included in this review are outlined in Table 1.

**Table 1:** Characteristics of the included studies with data on time of self-harm presentations to Emergency Departments

Author	Country	Study design	Source of data	Study time-frame	Sample age	Type of analysis	Self-harm inclusion criteria	Methods of self-harm	Times of self-harm presentations to EDs
Griffin et al. 2017 [19]	Ireland	Point-in-time data	National self-harm registry of Ireland	2007–2015	All ages	Descriptive analysis	Self-harm or ingestion of a substance in excess of the prescribed or recognised therapeutic dosage irrespective of motive	N/A	Presentations of self-harm on public holidays were most likely to occur out-of-hours between midnight and 09:00 am [males (1.15, 95% CI 1.04–1.27); females (1.35, 95% CI 1.20–1.51)]
Opmeer et al. 2017 [20]	United Kingdom	Retrospective before and after cohort study	Self-Harm Surveillance Register data from a large hospital in southwest England	1st January—31^st^ March both in 2014 and 2015	All ages	Descriptive analysis	Self-poisoning or self-injury irrespective of motive	Self-poisoning (72%), self-injury (16%), both of these (7%) and other/unknown (5%)—these are approximate percentages	20% of ED attendances in 2014 and 2015 occurred during the original Liaison Psychiatry Services (LPS) working hours (Monday–Friday, 09:00–17:00)
Kapur et al. 2008 [10]	United Kingdom	Point-in-time data	Six hospitals in Oxford, Leeds, and Manchester	1st March 2000 to 31st August 2001	All ages	Univariate logistic regression	Self-poisoning or self-injury irrespective of motive	Self-poisoning only (80%), self-poisoning and self-injury (4%), self-cutting only (13%) and other self-injury (3%). Self-poisoning here relates to drug overdoses	26.6% of self-harm presentations occurred during office hours (09:00–17:00); 49.6% occurred in evening hours (17:00–01:00); and 23.7% occurred in early morning (01:00–09:00)
Bergen & Hawton 2007 [21]	United Kingdom	Point-in-time data	Oxford Monitoring System: all individuals who presented to the general hospital in Oxford with self-harm	1997 to 2002	Persons aged 15 and older	Descriptive analysis	Intentional self-poisoning or self-injury, irrespective of motivation. Suicidal intent was measured using a 15-item measure	It does stratify between self-injuries and self-poisonings. The peak time for self-injury was midnight—04:00am. The peak time for self-poisonings was from 8:00 am—noon	The time with the highest rate was between 20:00–03:00. The peak time was 23:00–01:00. The lowest rate was between 04:00–10:00 after which the rate increased until the peak time. The majority (72%) occurred outside working hours. The authors did provide a breakdown for the three age groups: 15–19 years, 20–54 years and 55 + years. Peak hourly rates of presentation were between 23:00—midnight for adolescents aged 15–19 years (8.3%); between 23:00 and 01:00 for those aged 20–54 years (7.2%); and between 18:00 and 19:00 for those aged 55 + years (8.2%). Alcohol was strongly associated with the hour of presentation for self-harm both for the time of the self-harm act and in the six hours before the act (both used the chi-square test with *p* < 0.001). Self-harm presentations involving alcohol were most common between 20:00 and 08:00. With regards to suicidal intent, it was apparent that more patients with high intent presented in the evening hours (16:00 pm to midnight) than in the daytime hours (8:00 am to 16:00 pm). Higher suicidal intent was associated with males and the older age cohorts. Less than 30% of patients presenting outside the hours 8:00 am to 16:00 pm received a psychosocial assessment. The day with the highest amount of self-harm presentations was Sunday (15.6%), then Saturday (14.5%) and then Monday (14.7%)
Gunnell et al. 2006 [22]	United Kingdom	Point-in-time data	A stratified random sample of 32 hospitals was selected from a list of all general hospitals in England providing an accident and emergency (A&E) service	2001–2002	All ages	Descriptive analysis	Self-poisoning or self-injury irrespective of motive	Overdoses (79.4%), self-cutting (11.4%), overdoses and self-cutting (4.8%), and other methods (4.4%) including self-poisoning using non-medicinal products, attempted hanging, jumping and carbon dioxide poisoning	80% of self-harm presentations were outside working hours (9:00–17:00 Monday to Friday) and the peak time was 20:00–02:00. The peak day for females was Sunday and the peak day for males was Monday. Levels of those receiving a psychological assessment were slightly higher for those presenting during working hours compared to outside working hours
Nadkarni et al. 2000 [23]	United Kingdom	Retrospective case note study	The A&E department of the Leicester Royal Infirmary	Full year 1996	Children and adolescents	Descriptive analysis	Intentional self-injury (non-fatal), or deliberate ingestion of more than a prescribed amount of medical substances, or the deliberate ingestion of substances never intended for human consumption	92% was self-poisoning (with only one episode using an agent other than drugs). 9% was by wrist self-cutting	The highest percentage of self-harm presentations (37%) was between 18:00–midnight. More cases presented during Spring (30%) than during other seasons but there was no statistically significant variation between seasons. Highest numbers occurred during January, then April and then March. The lowest numbers occurred during February and then August. Most occurred during weekdays (70%) compared to weekends. 65% of self-harm events occurred at home/place of residence; 12% occurred in a non-school public place and 5% occurred in school
Caterino et al. 2013 [24]	United States of America	Prospective observational cohort study	Eight EDs participating in the ED-SAFE study (Convenience sample)	August 2010 – January 2012	Adults aged 18 +	Descriptive analysis	Suicide attempts, suicidal ideation, and non-suicidal self-injury	N/A	60% of self-harm presentations occurred between 07:00 and 15:00. 40% of self-harm presentations occurred between 15:00 – 07:00
McNicholas et al. 2009 [25]	Ireland	Retrospective case study	Case notes from Our Lady's Hospital for Sick Children, Crumlin, Dublin ED	1993–2003	Children and adolescents	Descriptive analysis	Self-poisoning or self-injury, irrespective of the apparent purpose of the act	N/A	80% presented outside normal hours (9:00–17:00 Monday—Friday) with 93% admitted for deliberate self-harm. Nearly all cases received a psychiatry assessment whether presenting within (96%) or outside (95%) of working hours
Byrne et al. 2011 [26]	Ireland	Retrospective case study	Case notes from Our Lady's Hospital for Sick Children, Crumlin, Dublin ED	2002–2008	Children and adolescents	Descriptive analysis	Mental health presentations (self-harm responsible for 58% of presentations, 27.8% for suicidal ideation—together 80%)	Of those who presented with self-harm, an overdose with medication was the most common method used	Only 41% of individuals presented between 9:00–17:00 and two-thirds presenting outside working hours. The highest rates of presentations occurred midweek and the lowest rates were at weekends
Arensman et al. 2018 [9]	Ireland	Point-in-time data	National self-harm registry of Ireland	2004–2012	All ages	Univariate analyses: multinomial logistic regression	A deliberate self-harm act with non-fatal outcome in which an individual deliberately initiates, or deliberately ingests a substance in excess of the prescribed dosage, and which is aimed at realising changes that a person desires via the actual or expected physical consequences	Drug overdose (67%), self-cutting (15%), drug overdose and self-cutting (4%), attempted hanging (3%), attempted drowning (2%), other methods (8%)	Time of admission to ED was split into the following six time-frames from most occurring to least occurring: 20:00–midnight (23.3%); midnight–03:59 (22.8%); 16:00–19:59 (19.4%); midday – 15:59 (15.3%); 04:00–07:59 (10.7%); 08:00–11:59 (8.5%). 40% involved alcohol. 30% of self-harm presentations were during the weekend
Hawton et al. 2007 [27]	United Kingdom	Point-in-time data	Data on self-harm presentations to general hospitals in Oxford (one hospital), Manchester (three hospitals) and Leeds (two hospitals), collected through monitoring systems in each centre	1st March 2000 to 31st August 2001	All ages	Descriptive analysis	Self-poisoning or self-injury irrespective of motive. Self-poisoning includes the intentional ingestion of more than the prescribed amount of any drug, whether or not there is evidence that the act was intended to result in death	Self-poisoning (80.8%), self-injury only (15%), both self-injury and self-poisoning (4.2%). The predominant method of self-injury was self-cutting. Other methods mentioned were attempted hanging, jumping from a height, inhalation of CO gas, attempted drowning, traffic related injury and head banging	The largest number of self-harm presentations occurred between 22:00 and 02:00 and the lowest number of presentations from occurred between 06:00 and 09:00. In nearly 55% of episodes of self-harm, the person had consumed alcohol within the six hours leading up to the act
Doshi et al. 2005 [28]	United States of America	4-stage probability sample of visits to non-institutional general and short-stay hospitals	National Hospital Ambulatory Medical Care Survey, a national probability sample of ED visits	1997–2001	All ages	Trend analyses	Attempted suicide and self-inflicted injury, defined by an International Classification of Diseases, Ninth Revision, Clinical Modification (ICD-9-CM) code of E950 to E959	Poisoning (68%), self-cutting (20%), attempted hanging (< 1%), self-inflicted injury using a firearm (< 1%)	The highest time for attempted suicide or self-inflicted injury appears from 18:00 to 22:00, with high rates from 18:00-midnight
Prescott et al. 2009 [29]	United Kingdom	Electronic patient records	Computerized admissions database known as the ED Information System (EDIS) using Crystal II database interrogation	1 April 2006 and 31 March 2007	All ages	Retrospective analyses	Specific to self-poisoning (ingestion of a substance in excess of the prescribed dosage, irrespective of motive)	Top three overdose substances used: paracetamol (42.5%), Ibuprofen (17.3%), Citalopram (6.7%)	Specific to self-poisoning self-harm presentations: most common time for these presentations to EDs was between 22:00 and 02:00. 70.7% of people presented to ED within 4 h of the self-poisoning
Blenkiron et al. 2000 [30]	United Kingdom	Prospective study of 158 adults referred for psychiatric following an episode of self-harm	York District Hospital	March to July 1997	All ages	Retrospective analyses	Self-harm according to the 10th edition of the International Classification of Diseases	15.2% of “early acts” of self-harm were not drug overdoses. 10.7% of “late acts” of self-harm were not drug overdoses	The times for the self-harm act were recorded, not the time at which they arrived in ED. The most common time for self-harm act was from 22:00 to midnight. The following two time-frames are compared: “early” 03:00 am–15:00 pm and “late” 15:00 pm to 03:00am. People whose act was “early” were more likely to be admitted to a medical ward than those whose act was “late” (70% versus 46%). Higher rates of alcohol use were recorded for the “late” acts than the “early acts (64.7% vs 39.4%). In terms of issues leading up to the act of self-harm, problems identified were relationships with partner/family, money, mental /physical health, work, lack of close friends, housing, alcohol/drugs or the death of someone close
Colman et al. 2004 [31]	Canada	Point-in-time data	The Ambulatory Care Classification System (ACCS), a database that tracks, among other information, all emergency department (ED) services provided in the province of Alberta	Three fiscal years 1998/9 to 2000/01	All ages (does stratify by age)	Descriptive analysis	ICD-9 Self-inflicted injury including self-poisoning	Self-poisoning was the most common method (81% for females and 71% for males); this was followed by self-cutting (14% for both genders), and then firearms/jumping/jumping/gas (2% for females and 7% for males)	The lowest rates for self-injury in adults were seen from November to February, and for children it was during the middle of summer The highest rates for adults were in May and for children in March Adults were most likely to present on Saturdays or Sundays, and for children it was Sundays or Mondays. Adults’ visits peaked in late morning (10:00 –midday), but were also high in the evening (20:00 –midnight). For children, there was a smaller rise in the number of visits in the late morning, with rates peaking at night (21:00–02:00)
Rhodes et al. 2008 [32]	Canada	Point-in-time data: descriptions of frequency data and missing data and cross-tabulations	Data from the National Ambulatory Care Reporting System (NACRS) were used to identify ED presentations to hospitals in Ontario, Canada	1st April 2001–31st March 2002	12–64-year olds	Descriptive analysis	Medicinal self-poisoning (part of deliberate self-harm)	N/A	This study was specific to deliberate self-poisoning self-harm presentations—78% were outside office hours
Perera et al. 2018 [14]	Australia	Retrospective point in time data	Linked ER data collection registry data for presentations to New South Wales public hospitals	2010–2014	All ages	Retrospective descriptive analysis	Mental health presentations including self-harm, suicidal ideation and intentional poisoning presentations	N/A	More than half of all presentations in these categories were outside normal working hours (08:00–18:00). Highest rate of self-harm presentations was between 18:00–midnight. 60% of presentations were described as urgent or potentially life-threatening
Subba et al. 2009 [15]	Nepal	Retrospective point in time data	Medicolegal register maintained by ED of the Western Regional Hospital	April 2002–March 2005	All ages	Retrospective descriptive analysis	Deliberate self-harm includes parasuicide and suicide	Self-poisoning (90%), hanging (7%), self-burning (2%), self-cutting (1%), drowning (1%)—approximate figures and two thirds of self-poisonings were by ingestion of organophosphate pesticides	The most common time for self-harm presentations occurred between May and July and in the last quarter of the day
Jegaraj et al. 2016 [16]	India	Retrospective observational study	ED of Christian Medical College, Vellore, India,	2011–2013	All ages	Descriptive analysis	Deliberate self-harm including suicide attempts and self-poisoning	Poisoning via agricultural chemicals (46%), tablet overdose (30%), plant poisons (8%), near hanging (5%), corrosive ingestion (5%), rat killer poison (4%)	37.1% of self-harm presentations occurred from 21:00 to 05:00 was the most common timeframe. June, May and September were the months with the highest self-harm presentations for the three years
Hickey et al. 2001 [3]	United Kingdom	Matched control design—the outcome of a group of patients who did and did not receive a psychiatric assessment	Oxford Monitoring System for Attempted Suicide	2-year period (Dates not mentioned)	All ages	Comparison between the control and study group using chi square test with Yate's correction and McNemar's corrected chi square test	Self-poisoning or self-injury irrespective of motive	In the assessed group, 49% was self-poisoning and 51% was self-injury In the non-assessed group, 75% was self-poisoning and 25% was self-injury	In the non-assessed group, 80.8% occurred during 17:00 –09:00 am and 19.2% between 9:00 am and 17:00. In the assessed group, 55% occurred during 17:00–09:00 and 45% between 09:00 and 17:00. In the non-assessed group, 27.6% occurred during weekends and 72.4% occurred during weekdays. In the assessed group, 20.8% occurred during weekends and 79.2% occurred during weekdays. 58.9% of self-harm patients discharged from ED did not have a psychiatric assessment. Patients with a self-harm presentation between 17:00 and 9:00 were less likely to get a psychiatric assessment. Non-assessed patients had a higher risk of further self-harm or completed suicide
Corcoran et al. 2015 [17]	Northern Ireland, UK	Point-in-time data	Tyrone County Hospital 2007–2010, Altnagelvin Hospital and Erne Hospital 2007–2012	2007—2012	All ages	Retrospective descriptive analysis	A deliberate self-harm act with non-fatal outcome in which an individual deliberately initiates, or deliberately ingests a substance in excess of the prescribed dosage, and which is aimed at realising changes that a person desire via the actual or expected physical consequences	70.4% involved drug overdoses. It was more common in women. Lethal methods were more common in men: attempted hanging (4.1% vs 1.4%) and attempted drowning (5.6% vs. 2.1%)	Alcohol was involved in 59.7% of self-harm presentations. This study stratified for men and women and for presentations involving alcohol and not involving alcohol. For both men and women, self-harm presentations involving alcohol were in excess on Sundays and Mondays, whereas there tended to be an even spread across the seven days for non-alcohol presentations. Alcohol self-harm presentations peaked from midnight to 05:00 whereas non-alcohol involved presentations roughly peaked (with far less of a peak) from 18:00 to midnight
Carroll et al. 2016 [18]	United Kingdom	Manchester Self-harm (MaSH) project	Three Hospitals in Manchester	2003–2010	16 years and older	Retrospective descriptive analysis	Self-poisoning or self-injury irrespective of motive	Self-poisoning (81.5%), self-injury (13.5%), other (5%)	A histogram indicated that most self-harm presentations were made from approximately 20:00–03:00. There was higher proportion of patients receiving a psychosocial assessment during working hours. It was found that psychosocial assessments reduce risk of repeat self-harm. The involvement of alcohol was higher in the 13:00 pm-05:00am presentations compared to the 05:00am-13:00 pm presentations (55% vs 50%)

### Characteristics of the studies

In terms of location, half of the 22 studies were based in the United Kingdom; four were in Ireland; two were in the United States; two were in Canada; and, one study was in Australia. Only two of the included studies were from low- or middle-income countries—namely Nepal and India [15, 16].

For the type of data used: nine of the studies used registry data that was available in their respective jurisdiction; seven studies collected data directly from two or more hospitals; and, six studies used data from one hospital. Retrospective descriptive data analysis was completed in all of the studies. In 15 of the studies, participants of all ages were considered and the findings were reported for the group as a whole. The remaining studies specified one age group to be studied. There were only two studies (Bergen and Hawton, and Colman et al. [21, 31]) that stratified their data into different age cohorts. Corcoran et al. stratified their data by gender and whether alcohol was involved in the self-harm presentation or not [17]. The study periods ranged from less than a year in the case of Blenkiron et al. [30], to ten years, in the case of McNicholas et al. [25].

Data on time of self-harm presentations were presented in various ways across the studies. In most of the studies, peak presentation times are only briefly mentioned in the text. Arensman et al. partitions 24-h periods into six four-hour time-frames and presents the peak presentation times in a table alongside other variables [9]. Caterino et al. also presents data in a table but groups the hours according to day shift (07:00–15:00) and evening/night shift (15:00–07:00)[24]. Corcoran et al. uses a trend graph to illustrate the peak times of self-harm presentations at EDs on a per hour basis [17]. Carroll et al. presents the hourly number of self-harm presentations data in a histogram [18]. Hickey et al. [3] compared patients who did and did not receive a psychiatric assessment after presenting at an ED with self-harm and reports the most common timeframe for each of these groups.

Most of the studies included in this review report on all self-harm presentations that occurred during the specified study period. The Griffin et al. study was an exception however—its focus is on self-harm presentations during public holidays only throughout their study period [19]. Blenkiron et al. reported on the times of the self-harm and not the time of presentation at ED per se but has still be included in the analysis for this review [30].

### Self-harm inclusion criteria

There were different definitions of self-harm defined in the different studies. The various definitions of self-harm are outlined in Table 1. The majority (12 out of 22) refer to self-harm as being either self-injury or self-poisoning, irrespective of the motivation (suicidal attempt or non-suicidal self-harm). While the Hawton et al. 2007 study also uses this definition, it also gives a definition for self-poisonings, namely as, “the intentional ingestion of more than the prescribed amount of any drug, whether or not there is evidence that the act was intended to result in death” [27]. Both Corcoran et al. and Arensman et al. used the definition for self-harm that is used by the National Suicide Research Foundation (NSRF) Ireland i.e. the Platt et al. 1992 definition: “an act with non-fatal outcome in which an individual deliberately initiates a non-habitual behaviour, that without intervention from others will cause self-harm, or deliberately ingests a substance in excess of the prescribed or generally recognised dosage, and which is aimed at realising changes that a person desires via the actual or expected physical consequences” [4, 9, 17]. Both the Doshi et al. study and the Colman et al. study used the definitions for self-harm/suicide attempt according to the International Classification of Diseases, Ninth Revision (ICD-9), whereas the Blenkiron et al. study used the ICD-10 [28, 30, 31]. Subba et al. referred to deliberate self-harm as including “parasuicide” and suicide [15].

Hence, most the studies (18 out of 22) focused on all methods of self-harm, including both self-injury and self-poisonings. There were four exceptions. There were two studies by Rhodes et al. and Prescott et al. that focused solely on presentations involving self-poisonings [29, 32]. In particular, the Rhodes et al. study examined self-poisoning events as defined by the ICD-9: drugs, medicinal and biological substances or toxic effects of substances chiefly nonmedicinal as to source [32]. Prescott et al. use the term “self-poisoning” while referring to overdosing with drugs, with a particular interest in paracetamol [29]. On the other hand, there were two more studies [14, 26] that focused on all mental health presentations made to an ED but self-harm made up the majority of presentations in these both of these two studies. Byrne et al. reported on mental health presentations to EDs although self-harm did make up the majority (58%) of presentations at EDs analysed in that study, followed by suicidal ideation presentations (27.8%) [26]. The Perera et al. study analysed mental health presentations to New South Wales EDs including self-harm, suicidal ideation or self-poisonings [14].

### Methods of self-harm

**Fig. 3:**
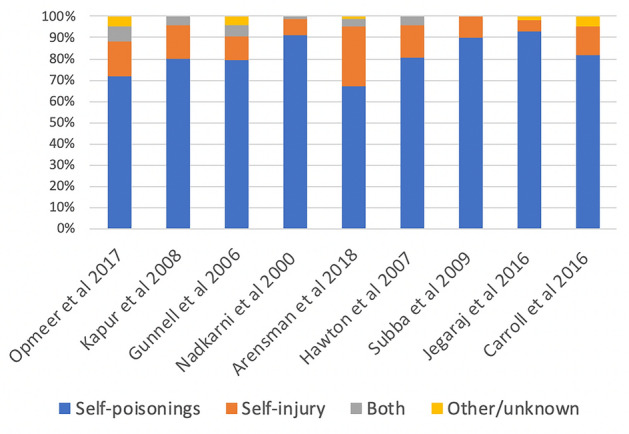
A percentage breakdown for self-injury versus self-poisonings in nine of the included studies

There were 17 out the included 22 studies that did include specific data relating to the methods of self-harm making up the presentations at EDs. Out of these 17 studies, the majority (13/17) demonstrated that self-poisonings or overdoses accounted for a significant majority of self-harm ED presentations, ranging from approximately 70–90%. Self-injury (most commonly in the form of self-cutting) made up approximately 10–20% of self-harm ED presentations. It was possible to extract an approximate breakdown of the percentages for the methods of self-harm in nine of the studies and this is presented in Fig. 3. The percentages in Fig. 3 are approximate since it was not always clear if self-poisonings were exclusive to overdoses or if they also included non-medicinal self-poisonings. What is clear, however, is that self-poisonings (overdoses and self-poisoning by other means) was the predominant method involved in self-harm presentations across the studies. The next method was usually self-cutting, followed by other methods of self-injury such as attempted hanging, attempted drowning, inhalation of carbon monoxide, jumping from a height, or traffic or firearm related self-harm injuries. For example, the Arensman et al. study breaks down the percentages as follows: drug overdose (67%), self-cutting (15%), drug overdose and self-cutting (4%), attempted hanging (3%), attempted drowning (2%), other method (8%) [9]. Most other studies that did report such data on methods of self-harm broadly matched this pattern. The Subba et al. study was an exception [15]. In this study, self-poisonings made up the majority (90%) of self-harm presentations, but this was followed by attempted hangings (7%), and self-burning (2%)—both more than self-cutting (1%) [15]. Interestingly, while for most studies self-poisoning was totally or mainly made up of medicinal overdoses, in the Subba et al. study, self-poisonings were two thirds from the ingestion of organophosphate pesticides [15]. This latter study from West Nepal is similar to the Jegaraj et al. study in India, which found that poisoning via agricultural chemicals made up the majority (46%) of self-harm presentations, followed by tablet overdose (30%), plant poisons (8%), near hanging (5%), corrosive ingestion (5%), and rat killer poison (4%) [16]. It should be noted that these two studies were the only ones that were based in middle-to-low income countries included in the review. Corcoran et al. found that more lethal methods were more common amongst men: 4.1% in males versus 1.4% in females for attempted hanging; and 5.6% in males versus 2.1% in females for attempted drowning [17].

The Bergen and Hawton study did not give the exact detail of the various methods of self-harm in the same way that these latter studies did; however, it did compare the times for self-injury versus self-poisonings [21]. These authors found that the peak time for self-injury was from midnight—04:00, whereas the peak time for self-poisonings was from 8:00 to noon [21]. Blenkiron et al. divided the day into two ranges: “early” from 03:00 to 15:00 and “late” from 15:00 to 03:00 [30]. This study found that 15.2% of “early acts” of self-harm were not drug overdoses and that 10.7% of “late acts” of self-harm were not drug overdoses [30].

With regards to suicidal intent, Bergen and Hawton reported that more patients with high intent presented in the evening hours (16:00 pm to midnight) than in the daytime hours (8:00 am to 16:00 pm) and that higher suicidal intent was associated with males and the older age cohorts [21]. In their study, Perera et al. mention that 60% of self-harm presentations were described as urgent or potentially life-threatening [14].

### Time of day for self-harm presentations

**Fig. 4:**
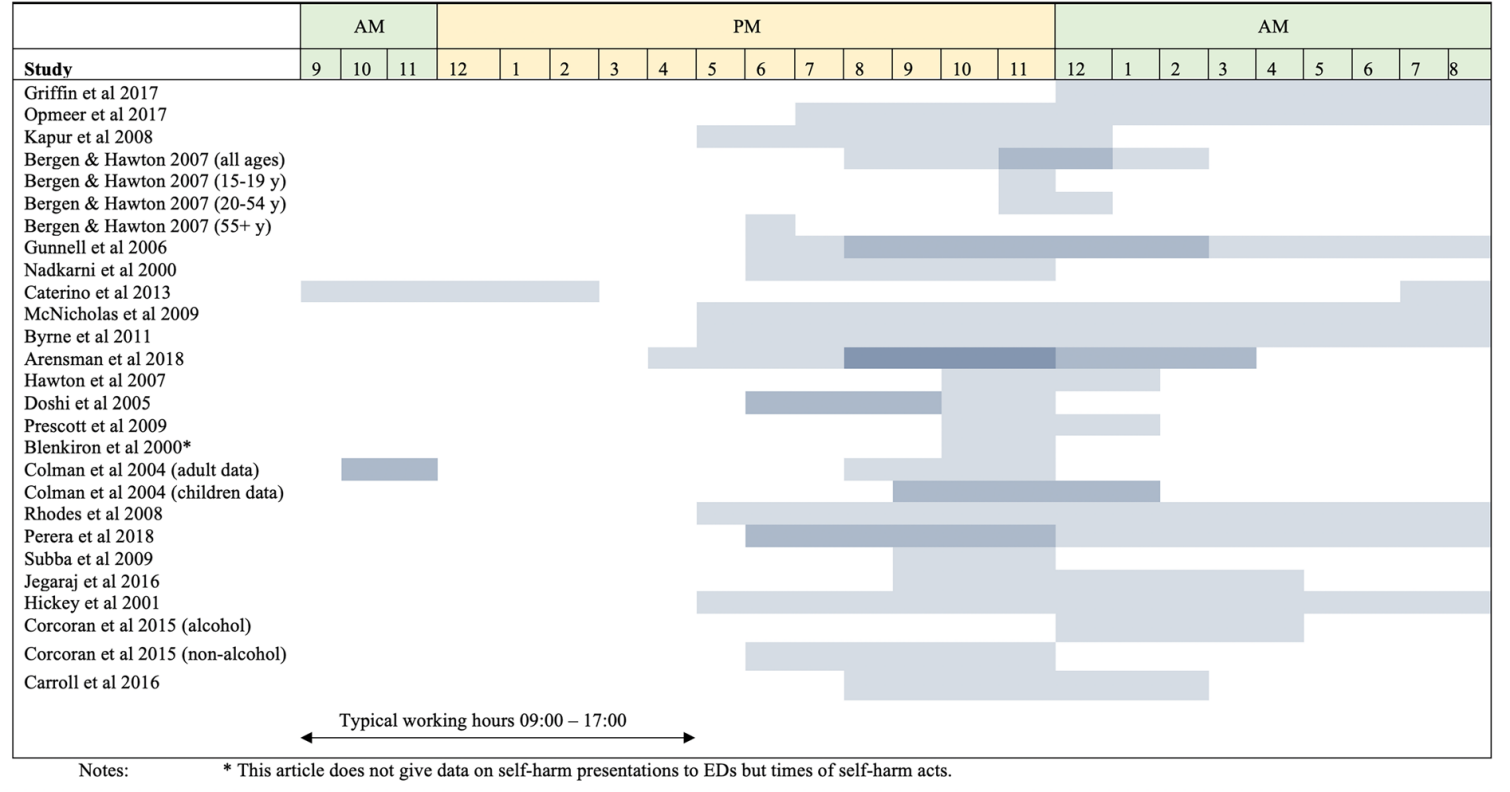
Peak times of self-harm presentations to EDs as reported by each study. (Darker colours represent times with higher frequency of presentations if this data was presented in the study)

In 20 out of the 22 included studies, the most common time frame for self-harm presentations to EDs was reported to be outside the normal office or working hours (Monday to Friday, 09:00–17:00). Figure 4 provides a visual presentation of the peak time frames reported across the studies. In six of the studies, a darker colour is used to highlight the most common time frame for self-harm presentations within that study. In three of the studies, some stratification of the data are displayed [17, 21, 31]. For the Hawton and Bergen study, the most common time frame for all ages is displayed (20:00 pm–03:00 am), followed by the most common times for the follow three age cohorts: adolescents (15–19-year olds), 20–54 year olds and 55 year olds and older (23:00-midnight, 23:00 pm–01:00 am, and 18:00 pm – 19:00 pm, respectively) [21]. Colman et al. also stratifies their data for age cohorts and this is displayed in Fig. 4 [31]. This study found that adults’ visits peaked in late morning (10:00 am –midday), but were also high in the evening (20:00 pm –midnight), whereas for children, there was a smaller rise in the number of visits in the late morning, with rates peaking at night (21:00 pm–02:00 am) [31].

The Corcoran et al. study was the third study to stratify their data but data were stratified according to whether alcohol was involved in the self-harm presentation or not, and not for age cohorts as in the latter two studies [17]. This is also displayed in Fig. 4. Self-harm presentations involving alcohol peaked from midnight to 05:00 am whereas non-alcohol-involved presentations roughly peaked (with far less of a peak) from 18:00 to midnight [17]. Bergen and Hawton also mentions that alcohol was strongly associated with the hour of presentation for self-harm both for the time of the self-harm act and in the six hours before the act (both used the chi-square test with *p* < 0.001) [21]. Furthermore, this study mentions that self-harm presentations involving alcohol were most common between 20:00 pm—08:00 am [21]. Arensman et al. mention that 40% of the self-harm presentations in their data involved alcohol [9]. The Hawton et al. study stated that in nearly 55% of episodes of self-harm, the person had consumed alcohol within the six hours leading up to the act [27]. Carroll et al. mentions that the involvement of alcohol was higher in the 13:00 pm—05:00 am presentations compared to the 05:00 am—13:00 pm presentations (55% vs 50%) [18]. Blenkiron et al. compared the following two time-frames: “early” 03:00–15:00 and “late” 15:00 to 03:00 [30]. This study found that higher rates of alcohol use were recorded for the “late” acts than the “early” acts (64.7% vs 39.4%) [30].

It appears that the timeframe from approximately 20:00 to 03:00 is the peak time for self-harm presentations at EDs across the majority of the studies. In the Arensman et al. study, the 20:00 pm to midnight time-frame and the midnight to 04:00 am time-frame were the most common time-frames with 23.3% and 22.8% of people presenting during these times, respectively [9]. This is also reflected by the different coloured shading in Fig. 4. The time-frames with the least occurring presentations were from 08:00 am to midday (8.5% of presentations) and from midday to 16:00 pm (15.3%) [9]. According to Gunnell et al. [22], Hawton et al. [27], Prescott et al. [29] and Jegaraj et al. [16], the most common timeframes for self-harm presentations at EDs are 20:00 pm–02:00 am, 22:00 pm–02:00 am, 22:00 pm-02:00 am and 21:00 pm-05:00 am, in their studies, respectively.

Other studies simply report that the majority of self-harm presentations occurred outside of typical working hours [14, 20, 25, 26, 32]. Opmeer et al. and McNicholas et al. reports this to represent 80% of presentations [20, 25]. Byrne et al. reports the majority to be two thirds [26]; Rhodes et al. reports 78% [32]; and, Perera et al. states that more than half of all self-harm presentations occurred outside of working hours [14].

As seen in Fig. 4, only two studies report peak presentations during typical working hours: namely, the Caterino et al. and the Colman et al. studies [24, 31]. In the first of these, based in the United States, Caterino et al. found that the majority (60%) of self-harm presentations occurred during working hours of 07:00–15:00 [24]. In the Colman et al. study, which stratified for adults and children, the children did peak outside working hours (21:00–02:00) but the adults peaked in the late morning (10:00–midday), but were also high in the evening (20:00 –midnight) [31].

### Most common days

As well as reporting on the most common time of day of presentations, seven studies also examined the most common day of the week. Colman et al. report that adults were most likely to present on Saturdays and Sundays, whereas children were most likely to present on Sundays or Mondays [31]. Corcoran et al. found that for both men and women, self-harm presentations involving alcohol were in excess on Sundays and Mondays, whereas there tended to be an even spread across the seven days for non-alcohol self-harm presentations [17]. Bergen and Hawton report that the day with the highest amount of self-harm presentations was Sunday (15.6%), followed by Saturday (14.5%) and then Monday (14.7%) [21]. Gunnell et al. report that the peak day for females was Sunday and the peak day for males was Monday [22]. Nadkarni et al. states that most presentations occurred during weekdays (70%) compared to weekends [23]. Arensman mentions that 30% of presentations occurred during the weekends [9]. Byrne et al. exclusively examined data for children and adolescents which may explain why it had different results in this regard: the highest rates of presentations occurred midweek and the lowest rates were at weekends [26].

### Seasonal trends

Seasonal trends were also examined in four studies and are included here as a secondary outcome. Subba et al. found that the most common time of year for self-harm presentations occurred between May and July [15]. Jegaraj et al. found a similar result with June, May and September being the months with the highest numbers of self-harm presentations for the three years [16]. In the Colman et al. study, they found that for adults, presentations were lowest between November to February while for children, they were lowest during the summer months [31]. Nadkarni et al. reported that more cases presented during spring (30%) than during other seasons but there was no statistically significant variation between seasons [23]. Moreover, this study mentions that the highest numbers occurred during January, then April and then March—with the lowest numbers occurred during February and then August [23]. Colman et al. states that the highest rates for adults were in May and for children in March [31].

### Circumstances of self-harm incident

There was limited information available about the circumstances of the self-harm episode that resulted in a presentation to the ED. For example, only one study reported on the timeframe between the actual time of self-harm and the subsequent ED presentation [29]. Prescott et al. reported that 70.7% of people who presented to ED with self-poisoning (mainly overdose) did so within 4 h of the self-poisoning [29]. The place at which the self-harm incident occurred was also just reported in one study where it was noted that 65% of self-harm events occurred at home/place of residence; 12% occurred in a non-school public place and 5% occurred in school [23]. Finally, in relation to circumstances that may have led to the self-harm act itself, Blenkiron et al. reported that problems with partner/family, money, mental /physical health, work, lack of close friends, housing, alcohol/drugs or the death of someone close as potential contributing factors [30].

### Follow-up data

There were also limited data on the treatment or referrals of patients who presented at EDs for self-harm. Most of the studies that include such data demonstrated higher levels of psychiatric assessment for those attending EDs during working hours. Blenkiron et al. compared the following two time-frames: “early” 03:00–15:00 and “late” 15:00–03:00 [30]. People whose act was “early” were more likely to be admitted to a medical ward than those whose act was “late” (70% versus 46%) [30]. Bergen and Hawton mention that less than 30% of patients presenting outside the hours 8:00–16:00 received a psychosocial assessment [21]. Similarly, Gunnell et al. found that levels of those receiving a psychological assessment were slightly higher for those presenting during working hours compared to outside working hours [22]. Hickey et al. also found that patients with a self-harm presentation between 17:00 and 9:00 were less likely to get a psychiatric assessment and that non-assessed patients had a higher risk of further self-harm or death by suicide [3]. They also found that 58.9% of self-harm patients discharged from ED did not have a psychiatric assessment [3]. Carroll et al. also found that there was a higher proportion of patients receiving a psychosocial assessment during working hours [18]. In contrast to all of these latter studies, the study by McNicholas et al. found that nearly all cases received a psychiatry assessment whether presenting within (96%) or outside (95%) of working hours [25]. This latter study was based in a children’s hospital.

## Discussion

This study sought to investigate the evidence regarding the most prevalent times at which people who have self-harmed (or had a suicide attempt) attended ED for treatment. The design of this study was a scoping review of the literature. While a satisfactory number of studies were included for data extraction, time of day of self-harm presentation was a secondary outcome across most studies. Most studies also focused on adult samples and did not stratify the data by age cohorts. The evidence from the data extraction for this review is that self-harm presentations tend to be highest at EDs during weekends and outside of office working hours of 09:00–17:00, Monday to Friday. In particular, they tend to occur most frequently in the three hours before and after midnight.

Many conjectures could be made to explain why self-harm presentations tend to most frequently occur outside office working hours but alcohol use (and possibly drug use) in evening times and during weekends appears to be a crucial factor to consider. Griffin et al. found that time of presentation at EDs was associated with alcohol being involved and that this association was stronger in women [34]. That study also found that presentations that occurred between midnight and 09:00 were most likely to involve alcohol [34]. Corcoran et al. found that there was a continued increase in alcohol-related self-harm presentations for both men and women during evening hours with a peak in the early hours of the morning [17]. Carroll et al. and Blenkiron et al. also found that later self-harm presentations were associated with alcohol consumption [18, 30]. Hence, the use of alcohol could be a prominent factor leading to higher numbers of self-harm presentations to EDs outside office working hours. Indeed, Corcoran et al. found that alcohol was involved in 59.7% of self-harm presentations at EDs in their study of three hospitals in Northern Ireland [17].

It could also be hypothesised that some patients waited to present to EDs during the night to encounter fewer staff and to decrease the chances of being seen entering the hospital out of shame or fear of stigmatisation. Indeed, Professor Rory O’Connor refers to suicide as “one of the last remaining taboos” and calls it “the big S”—similar to how cancer (“the big C”) was taboo 20–30 years ago [35]. The same could be said of self-harm. In a systematic review by Hepp et al. [36], one study [37] found that urges for self-harm peaked at 15:00 whereas another study [38] found they were most common during the evening times. Blenkiron et al. was the only study included in this review that reported a time of the self-harm act and not the time of presentation to ED and it reported 22:00—midnight as being the most common time frame [30]. While there may be distinctions as to when the self-harm act occurred and when the time of presentation at ED occurred, the Prescott et al. study reported that 70.7% of people who presented to ED with a self-harm episode (in this case, specifically self-poisoning) did so within 4 h of the self-poisoning [29]. In their study, Perera et al. mention that 60% of self-harm presentations were described as urgent or potentially life-threatening; hence, it could be deduced that most self-harm presentations occurred within a short time frame of the self-harm act itself [14]. Thus, while there are limited data on the self-harm episodes prior to the self-harm presentations at EDs, it appears that the acts themselves mostly tend to also occur in the evening times and within a few hours of the self-harm presentations, which we know tend to occur in the evening hours also and outside working hours.

Altogether, it may be impossible to determine why exactly most self-harm presentations occur at EDs outside working hours. Self-harm, like suicide is a multifaceted phenomenon. As O’Connor mentions, suicide is not caused by a single factor; rather suicide (and self-harm) results from a perfect storm of factors and these can be biological, psychological, clinical, social, cultural and many of them may be hidden [35]. Blenkiron et al. mentions other factors that were associated with self-harm presentations; namely, problems with partner/family, money, mental /physical health, work, lack of close friends, housing, alcohol/drugs or the death of someone close [30]. Hence, while alcohol may be a prominent factor involved in self-harm presentations, it is most likely that its combination with other factors is what is important. There were seven studies that reported on the most common days for self-harm presentations for adults and it is likely that alcohol contributed greatly to weekends and Mondays usually being the most common days, with presumably more alcohol being consumed during the weekends.

There were two studies that reported that the peak times for self-harm presentations at EDs were during working hours, namely the Caterino et al. and the Colman et al. studies [24, 31]. There are no data given in these studies to indicate why they were different. The Colman et al. study does mention that 10:00—midday was the peak time for adults but it also mentions that the rates were high from 20:00 to midnight for adults [31]. Moreover, it states that for children, the peak times was 21:00–02:00, similar to most other studies [31]. Hence, while it does have a different adult peak time, the results are not in stark contrast to the other 20 studies that reported to have the peak time outside working hours. The Caterino et al. study chooses unusual times to spilt the 24 h clock: it reports that 60% of presentations occurred during the 7:00–15:00 time frame and 40% of presentations occurred during the 15:00–7:00 timeframe [24]. There is no further discussion on these times in the papers and any reasons mentioned here would be purely speculative. Despite these two studies, the results overall from this review do indicate that most self-harm presentations occur outside the usual working hours.

As well as the most common day for self-harm presentations, the most common seasons for self-harm presentations was another secondary outcome in this review (reported in only four studies). There was some limited evidence to suggest that self-harm presentations occur more frequently during summer months than in other seasons but this should be interpreted with caution. Three out of four studies indicated that there were higher numbers of presentations during the summer [15, 16, 31]. Two further studies, not included in this review, concur with this seasonal trend and are also from northern hemisphere countries. Simsek et al. found that May was the month with the highest numbers of self-harm presentations in its Turkish study [39]. Mejías-Martín et al., which examined emergency calls relating to self-harm (rather than ED data) from 2007 to 2013 in Andalusia, Spain, found that calls were most frequent during the summer months [40]. On the other hand, Nadkarni did report that Spring was the season with the highest self-harm presentations but it also reported that there was no statistically significant difference between the seasons [23]. Further international data is needed to determine if summer is indeed the season with the highest number of self-harm presentations. If it is, then it may be possible that alcohol may be a contributing factor to these observations but factors such as longer days, higher temperatures, idleness, or loneliness during the holiday season should also be considered.

Only two studies [21, 31] in this review stratified their data by age cohorts and only one of these reported on the most common time of year for self-harm presentations. In the Colman et al. study, a different seasonal trend existed for children when compared with other age cohorts [31]. Colman et al. found that self-harm presentation rates for children were in fact lowest during the summer months and that Sundays and Mondays were the most common days for these presentations [31]. One could speculate that this may be due to stress or anxiety associated with school. If this is the case, then the school environment, while not being attributed here as a causal factor, should at least be considered in the context of self-harm in children.

It is important for health service managers to be aware of the most common times at which presentations for self-harm (suicide attempts included) occurs in EDs so that the appropriate provision of available staff and services can be provided during the relevant timeframes. Kapur et al. recommend that all patients presenting at EDs with self-harm should receive a psychosocial assessment but there are wide variations between hospitals with many patients not receiving an assessment [9]. One of the possible determinants for patients not receiving such an assessment may be a lack of services outside usual office working hours. For example, Hickey et al. found that patients with a self-harm presentation between 17:00 and 9:00 were less likely to get a psychiatric assessment and that non-assessed patients had a higher risk of further self-harm or suicide [3]. Four other studies found a similar result [18, 21, 22, 30]. Conversely, McNicholas et al. reported that nearly all cases received a psychiatry assessment whether presenting within (96%) or outside of (95%) working hours but this study focused on children and adolescents, which may explain why it had different results [25]. Furthermore, the provision of different services is most likely to be different depending on the setting; whether a patient presenting with self-harm receives a psychiatric assessment or not may well be hospital dependent. Ultimately, the necessary resources (in particular, the provision of those able to conduct psychiatric assessments) should be made available outside of typical working hours to ensure the needs of self-harm patients are addressed. This may reduce the numbers of patients re-presenting at EDs with self-harm or, indeed it may help to prevent these patients from dying by suicide in the future.

One of the findings from this scoping review was that most of the studies did not stratify their data for age in the way that the Colman et al. study and the Bergen and Hawton study did [21, 31]. Indeed, both of these studies demonstrated different results for different age cohorts. This is an important finding for future research in this area. Given the wider age band for adult samples in comparison to adolescents, it is likely that data related to adults dominated the findings in many of the studies. This may have resulted in trends for other age cohorts, like children or adolescents, being hidden. It would be interesting to have seen the other authors stratify their data, in the way that the latter two studies [21, 31] did, by age and gender to see if there were any similar patterns; that is, if there was any evidence to suggest that self-harm rates for children were lower during the summer months. In terms of future research, more data analysis should be conducted on self-harm presentations to EDs involving age stratifications for cohorts like children, adolescents and adults.

Another important result from this scoping review is the fact that “time of self-harm presentations at EDs” tended not to be a primary outcome for most of the studies. Hence, any further review, especially in the case of a future systematic review on this topic would need to consider this when completing a keyword search for this data. The keyword search used in this scoping review used terms relating to “time of presentation”, “self-harm” and “emergency department” (See Fig. 1). This was most likely too specific since this keyword search did not pick up the six additional articles added during the search process [3, 14–18]. It would be important for future studies to consider this in their search process, since there may be other studies that do not mention time of presentation in the article title and abstract but do mention it as a secondary outcome in the full text of the article. However, given that this is a scoping review, highlighting that the primary outcome of this study was rarely a primary outcome in the included studies for data extraction is an important result in itself. For future related systematic reviews on this topic, it is recommended that a more general keyword search strategy be used and a thorough search is employed with full-paper screening of all relevant articles. In particular, it may be helpful to just use terms involving “self-harm” and “emergency department” but exclude terms relating to “time of presentation” since it is too specific.

It is also worth noting that this study was dominated by studies from western or high-income countries. There were only two exceptions from India and Nepal [15, 16]. Suicide and self-harm are culturally determined. Therefore, the results from this study should be considered to apply to a western society context. Future research projects should be completed using data from low- and middle-income countries.

The high quality of the data obtained in many of the included studies is a strength of this review. For example, three studies report on data collected by well-established surveillance systems in the UK and Ireland. Arensman et al. used NSHRI data of all self-harm presentations to every ED in the Republic of Ireland from 2004 to 2012 [9]. Hickey et al. and Bergen and Hawton both used data from the Oxford Surveillance System, which collects data relating to self-harm at the Oxford General Hospital [3, 21]. The majority of studies use comprehensive, high-quality data. Given that the outcome are times at which self-harm presentations are made to EDs and given the complete coverage of these events in hospital data implies that the results are most likely to be accurate and well-defined.

## Conclusion

The overwhelming evidence from this review is that self-harm presentations to EDs tend to occur outside office working hours 09:00–17:00, Monday to Friday. Hence, the provision of available staff and services must be provided for such presentations outside of normal working hours. Hospitals should employ robust surveillance systems to study the peak times, days and months for self-harm presentations and ensure that the adequate services are then available when they are needed the most. There were also some limited data to suggest that, for adults, self-harm presentations peak during the summer months, whereas, for children, they are lowest during this season. Further research is needed, however, to verify this finding, since it was only a secondary outcome for this review. Furthermore, future research projects studying self-harm presentations at EDs should stratify its data for different age cohorts and more data analysis is needed on self-harm presentations at EDs in low- and middle-income countries since the majority of included articles in this review were from high-income countries.

## Data Availability

Not applicable.

## Abbreviations

ED: Emergency department
ICD: International classification of diseases
NSHRI: National self-harm registry of Ireland
NSRF: National suicide research foundation (of Ireland)

## Declarations

### Patient and public involvement

No patient and public involvement.
